# Recurrence-Associated Multi-RNA Signature to Predict Disease-Free Survival for Ovarian Cancer Patients

**DOI:** 10.1155/2020/1618527

**Published:** 2020-02-14

**Authors:** Yu Zhang, Qingjian Ye, Junxian He, Peigen Chen, Jing Wan, Jing Li, Yuebo Yang, Xiaomao Li

**Affiliations:** Department of Gynecology, The Third Affiliated Hospital of Sun Yat-Sen University, Guangzhou 510630, China

## Abstract

Ovarian cancer (OvCa) is an intractable gynecological malignancy due to the high recurrence rate. Several molecular biomarkers have been previously screened for early identifying patients with a high recurrence risk and poor prognosis. However, all the known studies focused on a single type of RNAs, not integrating various types. This study was to construct a new multi-RNA-based model to predict the recurrence and prognosis for OvCa patients by using the messenger RNA (mRNA, including long noncoding RNA (lncRNA)) and microRNA (miRNA) sequencing data of The Cancer Genome Atlas database. After univariate Cox regression and least absolute shrinkage and selection operator analyses, a multi-RNA-based signature (2 miRNAs: hsa-miR-508, hsa-miR-506; 1 lncRNA: TM4SF1-AS1; 11 mRNAs: MAGI3, SLAMF7, GLI2, PDK1, ARID3A, PLEKHG4B, TNFAIP8L3, C1QTNF3, NDUFAF1, CH25H, TMEM129) was generated and used to establish a risk score model. The high- and low-risk patients classified by the median risk score exhibited significantly different recurrence risks (89% versus 61%, *p* < 0.001) and survival time (the area under the receiver operating characteristic curve (AUC) = 0.901 for 5-year disease-free survival (DFS)). This risk model was independent of other clinical features and superior to pathologic staging for DFS prediction (AUC, 0.906 versus 0.524; C-index, 0.633 versus 0.510). Furthermore, some new interaction axes were revealed to explain the possible functions of these RNAs (competing endogenous RNA: TM4SF1-AS1-miR-186-STEAP2, LINC00536-miR-508-STEAP2, LINC00475-miR-506-TMEM129; coexpression: LINC00598-PLEKHG4B). In conclusion, this multi-RNA-based risk model may be clinically useful to stratify OvCa patients with different recurrence risks and survival outcomes and included RNAs may be potential therapeutic targets.

## 1. Introduction

Ovarian cancer (OvCa) is one of the frequently diagnosed, lethal malignancies in the female genital system worldwide. It was estimated that there were 22,240 new cases and 14,070 deaths in the United States in 2018 [1] while 52,100 new cases and 22,500 mortalities were reported in China in 2015 [2]. Surgical resection combined with chemotherapy is the first-line treatment for OvCa, which has been demonstrated to improve the prognosis of patients. However, the overall five-year survival rate remains lower (approximately 30%) [3, 4] because more than 70% of patients [5] may experience recurrence, ultimately resulting in the treatment failure. Therefore, early identification of patients with a high recurrence risk is necessary in order to schedule personalized therapies and improve prognostic outcomes.

Recently, with the rapid developments in molecular biology, several scholars devote themselves to identify molecular biomarkers for prediction of recurrence and survival for OvCa patients. For example, Yang et al. used the microarray data of GSE9891 and GSE30161 retrieved from the Gene Expression Omnibus (GEO) database and LASSO (least absolute shrinkage and selection operator) penalized regression analysis to identify a six-long noncoding RNA (lncRNA: including RUNX1-IT1, MALAT1, H19, HOTAIRM1, LOC100190986, and AL132709.8) recurrence signature. This signature was found to predict the disease-free survival (DFS) for OvCa patients, with the area under the receiver operating characteristic (ROC) curve (AUC) of 0.813 (GSE9891, training), 0.697 (GSE9891, internal validation), and 0.711 (GSE30161, external validation) [6]. Dong et al. applied the support vector machine (SVM) method to screen a 19-microRNA (miRNA) signature that could well distinguish the recurrent from the nonrecurrent samples. Six of them (miR-193b, miR-211, miR-218, miR-505, miR-508, and miR-514) were found to be independently related to prognosis and were used to establish a risk score which was proved to significantly discriminate the recurrence-free survival (RFS) between the high-risk and low-risk groups (AUC = 0.961 for The Cancer Genome Atlas (TCGA) sequencing dataset; AUC = 0.922 for GSE25204 microarray dataset; AUC = 0.921 for GSE27290 microarray dataset) [7]. Zhou et al. also utilized the SVM algorithm to screen 39 feature genes. They distinguished the recurrence samples from nonrecurrence samples, with the prediction accuracies of 92.7%, 93.3%, 96.6%, and 90.4% for GSE17260, GSE44104, GSE51088, and TCGA datasets, respectively. The Kaplan–Meier (K-M) survival curve revealed that the survival time of patients with predicted nonrecurrent OvCa was significantly longer than that of patients with predicted recurrent OvCa [8]. Zhao et al. screened KCNN4 and S100A14 combination as the perfect recurrence prediction model (AUC = 0.5442–0.9524) [9]. However, all these studies focused on the prognostic values of single RNA type, not a combination of them [10]. The study on colorectal cancer indicated that a multi-RNA-based classifier model (AUC = 0.83) seemed to be more effective in stratifying patients who are at a high risk of mortality than lncRNAs (AUC = 0.56, *p* < 0.001), miRNAs (AUC = 0.67, *p*=0.0291), or mRNAs alone (AUC = 0.76, *p*=0.0051) [11]. Therefore, it is of clinical significance to develop a multi-RNA-based prognostic signature for OvCa.

In this study, we aimed to collect the lncRNA, miRNA, and mRNA expression data from the TCGA database and construct a multi-RNA-based risk score model to predict the recurrence and DFS for OvCa patients. In addition, the underlying functions of lncRNAs, miRNAs, and mRNAs were predicted by constructing the coexpression, competing endogenous RNA (ceRNA) [12], and protein-protein interaction (PPI) [8] networks, which was also not simultaneously analyzed in previous studies on the recurrence signature of OvCa. Accordingly, we hypothesize that our study may provide a more effective, function-clear signature for the prediction of recurrent prognosis in OvCa patients.

## 2. Materials and Methods

### 2.1. Patient Datasets

The mRNA (including lncRNA, *n* = 379) and miRNA (*n* = 498) expression data (level 3) with their clinical information were collected by searching the TCGA database portal (https://portal.gdc.cancer.gov/) on August 6, 2019, with the keyword of ovarian cancer. These expression profiles were obtained by sequencing on the Illumina HiSeq 2000 platform. A total of 322 samples were retained for the following analysis because they satisfied the following inclusion criteria: (1) mRNAs and miRNAs were both sequenced; (2) information of recurrence (*n* = 80, nonrecurrent; *n* = 242, recurrent) and survival status was clearly described. Due to the fact that no other datasets simultaneously met the above two inclusion criteria, these 322 patients, obtained from the TCGA database, were randomly divided to training and validation sets to achieve internal validation. Their detailed clinical characteristics are presented in Table 1.

### 2.2. Differential Expression Analyses

The mRNAs, lncRNAs, and miRNAs in RNA-seq profiles were annotated according to the annotation information of the HUGO Gene Nomenclature Committee (HGNC; http://www.genenames.org/) that includes 4,422 lncRNAs, 19,223 protein-coding genes, and 1,914 miRNAs [13]. The differentially expressed genes (DEGs), lncRNAs (DELs), and miRNAs (DEMs) between recurrent and nonrecurrent samples were identified using the Linear Models for Microarray Data (LIMMA) method (version 3.34.7; https://bioconductor.org/packages/release/bioc/html/limma.html) [14] in *R* package (version 3.4.1; http://www.R-project.org/). RNAs with false discovery rate (FDR) < 0.05 and |logFC(fold change)| > 0.5 were considered to be significant. Heat map of differentially expressed RNAs (DERs) was plotted with “pheatmap” package (version: 1.0.8; https://cran.r-project.org/web/packages/pheatmap) based on centered Pearson's correlation.

### 2.3. Construction of Multi-RNA-Based Prognostic Signature

Univariate Cox regression analysis was first performed to preliminarily screen the DEGs, DELs, and DEMs that were associated with DFS using the “survival” package in *R* (version, 2.41-1; http://bioconductor.org/packages/survivalr/). Subsequently, a multivariate Cox regression analysis was conducted to confirm their independence. Log-rank *p* value < 0.05 was set as the statistical threshold for these two analyses. In order to further optimize the prognostic signature, an L1-penalized estimation-based Cox proportional hazards regression model (that is, LASSO) in the penalized package (version, 0.9-5; http://bioconductor.org/packages/penalized/) [15, 16] was applied for the independent prognostic DERs, in which the value of penalty parameter lambda was chosen via cross-validation likelihood routine (1000 iterations). Then, the prognostic risk score was established according to the expression levels of the RNAs (Exp_DERs_) and prognostic coefficients (∑*β*_DERs_):

(1) $$\begin{array}{c} \text{Prognostic risk score}=\sum \beta_{\text{DERs}}\times {\text{Exp}}_{\text{DERs}}. \end{array}$$

### 2.4. Assessment of the Prognostic Performance of the Risk Score Model

Using the median risk score as the cutoff point, the OvCa patients were classified into high-risk and low-risk groups. K-M survival curve was drawn by using the “survival” package in *R* to observe the DFS differences between high- and low-risk groups. Furthermore, the ROC curves with the calculation of AUC were also plotted using the pROC in *R* (version, 1.14.0; https://cran.r-project.org/web/packages/pROC/index.html) to estimate the prediction accuracy of this risk score for the 1-, 3-, and 5-year survival rate of high- and low-risk patients. These analyses were performed for each dataset, respectively.

To verify that the multi-RNA signature was an independent prognostic factor, univariate and multivariate Cox regression analyses were performed for DFS using the risk score and several clinical features (including age, neoplasm histologic grade, pathologic stage, lymphovascular invasion, and vascular invasion) in each dataset. The superiority of the risk score model to clinical factors in survival prediction was also validated by K-M curve analysis for subgroups, calculation of AUC, and Harrell's concordance index (C-index) for each classifier. C-index was calculated using survcomp package in *R* (http://www.bioconductor.org/packages/release/bioc/html/survcomp.html) [17].

### 2.5. Function Prediction of the Prognostic RNAs

Coexpression, ceRNA, and PPI networks were constructed to predict the possible functions of signature lncRNAs, miRNAs, and mRNAs, respectively. Pearson correlation coefficients (PCC) between DELs and DEGs were calculated to represent their possible coexpression relationships using cor.test function (https://stat.ethz.ch/R-manual/R-devel/library/stats/html/cor.test.html) in *R*. Potential interactions between DEMs and DELs as well as between DEMs and DEGs were predicted using DIANA-LncBase (version, 2.0; http://carolina.imis.athena-innovation.gr/diana_tools/web/index.php?r=lncbasev2/index-predicted) [18] and starBase database (version, 2.0; http://starbase.sysu.edu.cn/) [19], respectively. Only the DEL-DEM and DEM-DEG interactors that had an opposite expression trend were used to construct the ceRNA network [20]. The interactions between DEGs were predicted by mapping the genes to the Search Tool for the Retrieval of Interacting Genes (STRING; version, 10.0; http://string-db.org/) database [21]. PPI network was constructed based on interaction pairs with a combined score >0.4. All networks were visualized using Cytoscape (version 3.6.1; http://www.cytoscape.org/).

Function enrichment analysis was performed for the genes in all three networks using the Enrichr online tool (http://amp.pharm.mssm.edu/Enrichr/) [22], in which Kyoto Encyclopedia of Genes and Genomes (KEGG), BioCarta, Reactome Pathways and Gene Ontology (GO) biological process (BP), and molecular function (MF) terms could be obtained. A *p* value < 0.05 was considered statistically significant.

## 3. Results

### 3.1. Differential Expression Analysis

**Figure 1:**
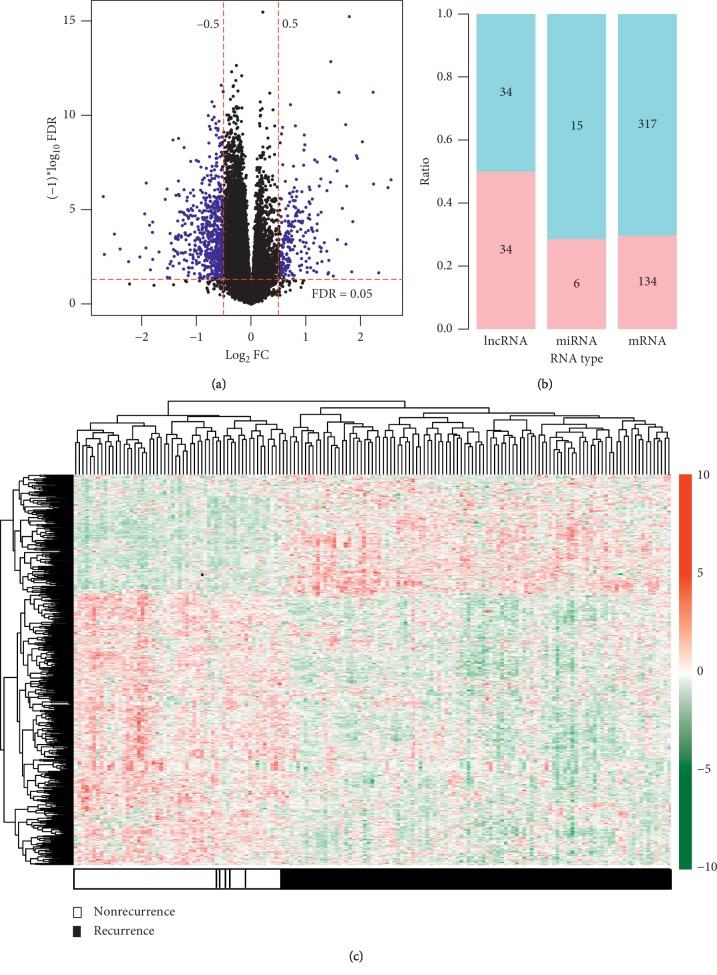
Differentially expressed RNAs. (a) Scatter diagram to show the distribution of differentially expressed RNAs (blue dot). The horizontal dotted line is FDR < 0.05; the vertical dotted line is |logFC| >0.5; (b) upregulated and downregulated number and ratio of each RNA type; (c) heat map of all differentially expressed RNAs. The horizontal axis is the sample, and the vertical axis is the expression level. Red, high expression; green, low expression. FC, fold change; FDR, false discovery rate.

A total of 12,728 mRNAs, 1,214 lncRNAs, and 547 miRNAs were annotated based on HGNC. Under the threshold of |log2FC| > 0.5 and FDR < 0.05, 540 of them were screened as DERs between recurrent and nonrecurrent samples (Figure 1(a)), including 451 DEGs, 68 DELs, and 21 DEMs (Figure 1(b)). The heat map showed that these DERs can obviously distinguish the recurrent from the nonrecurrent samples (Figure 1(c)).

### 3.2. Construction of a Prognostic Signature

**Figure 2:**
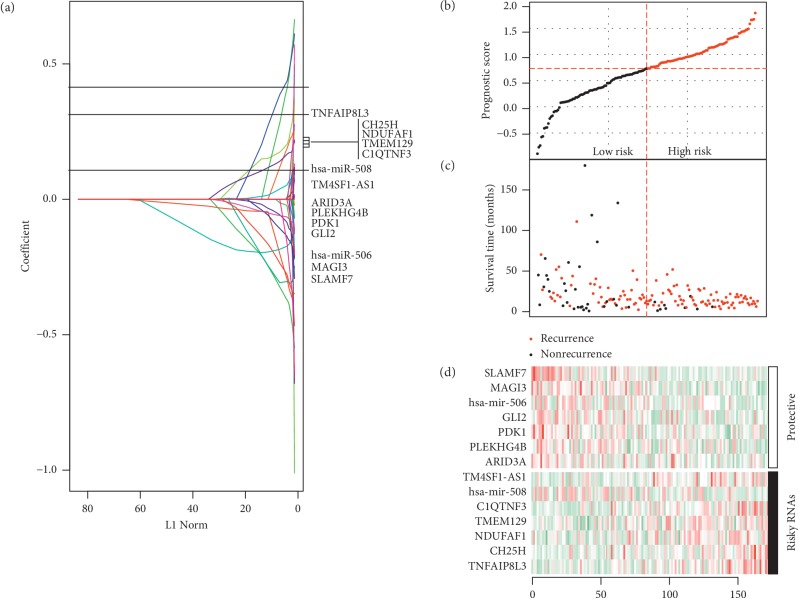
Construction of multi-RNA-based risk score model. (a) LASSO coefficient profiles of 14 survival-related RNAs; (b) the distribution of risk score in each patient in the training dataset; (c) the outcome of recurrence status and survival time. The red dotted line represents the optimum cutoff dividing patients into low-risk and high-risk groups; (d) heat map of the RNAs in the prognostic model.

Univariate Cox regression analysis identified 160 DERs to be significantly associated with DFS, including 140 DEGs, 10 DELs, and 10 DEMs. Multivariate Cox regression analysis filtered out 61 DERs as independent prognostic biomarkers, including 53 DEGs, 4 DELs, and 4 DEMs. Fourteen of them (2 DEMs: hsa-miR-508, hsa-miR-506; 1 DEL: TM4SF1-AS1; 11 DEGs: MAGI3, SLAMF7, GLI2, PDK1, ARID3A, PLEKHG4B, TNFAIP8L3, C1QTNF3, NDUFAF1, CH25H, TMEM129) were further selected as the optimal prognostic combination after LASSO analysis (Table 2; Figure 2(a)). Also, the results of the expression, univariate, and LASSO analyses were consistent, showing that hsa-miR-506, MAGI3, SLAMF7, GLI2, PDK1, ARID3A, and PLEKHG4B may be tumor suppressor genes and high expression of them predicted excellent prognosis while hsa-miR-508, TM4SF1-AS1, TNFAIP8L3, C1QTNF3, NDUFAF1, CH25H, and TMEM129 may be oncogenic genes and poor prognosis would occur in patients with high levels of them (Table 2). The prognostic risk score model was then established based on the expression of these biomarkers and their LASSO coefficients (Table 2; Figure 2(a)): prognostic risk score = (−0.20 *∗* hsa-miR-506) + (−0.27 *∗* MAGI3) + (−0.29 *∗* SLAMF7) + (−0.12 *∗* GLI2) + (−0.08  *∗* PDK1) + (−0.02 *∗* ARID3A) + (−0.03 *∗* PLEKHG4B) + (0.12 *∗* hsa-miR-508) + (0.04 *∗* TM4SF1-AS1) + (0.29 *∗* TNFAIP8L3) + (0.13 *∗* C1QTNF3) + (0.15 *∗* NDUFAF1) + (0.16 *∗* CH25H) + (0.14 *∗* TMEM129).

### 3.3. Assessment of the Prognostic Performance of This Signature

The risk score was calculated for each patient in each dataset (Supplemental [Supplementary-material supplementary-material-1]), and the patients in each dataset were divided into a high-risk group and low-risk group according to their median risk score. As shown in Figure 2(b), the patients with high-risk scores were shown to be at a high risk of recurrence (72/81, 89% versus 49/80, 61%; Chi-square = 16.5, *p* < 0.001) and had shorter DFS than those with the low-risk scores (Figure 2(c)). Also, patients with high-risk scores tended to express oncogenic RNAs, whereas tumor suppressor RNAs inclined to be highly expressed in patients with low-risk scores (Figure 2(d)).

**Figure 3:**
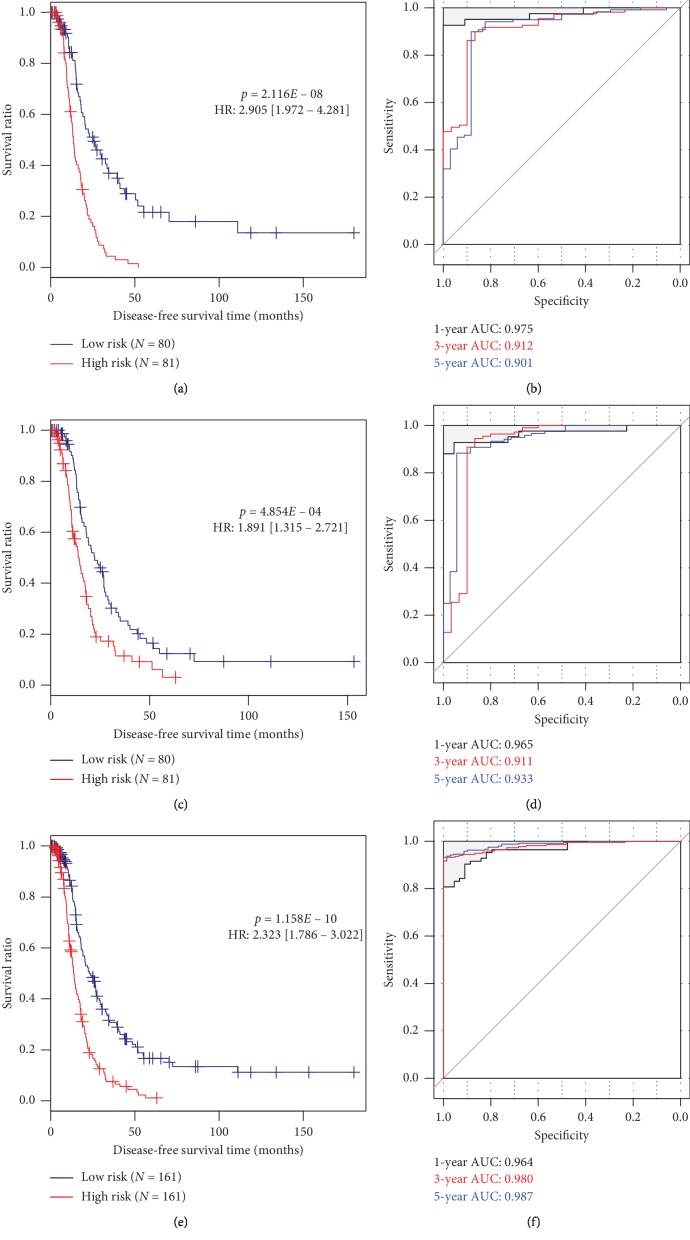
Prognostic performance assessment of the multi-RNA-based risk score model (a, c, e). ROC curves for the training (a), validation (c), and entire (e) datasets; (b, d, f) K–M curves for the training (b), validation (d), and entire (f) datasets. ROC, receiver operator characteristic; AUC, area under the curve; K–M, Kaplan–Meier; HR, hazard ratio.

To further confirm the prognostic performance of this signature, the K-M survival curve was drawn for the training (Figure 3(a)), validation (Figure 3(c)), and entire sets (Figure 3(e)). As expected, the DFS of the patients in the high-risk group was significantly shorter than that of the low-risk group in all sets. ROC curve analysis also validated that the predictive accuracy of this signature seemed to be relatively high, with the AUC of 0.901, 0.933, and 0.987 for the 5-year survival rate in the training (Figure 3(b)), validation (Figure 3(d)), and entire sets (Figure 3(f)), respectively.

**Figure 4:**
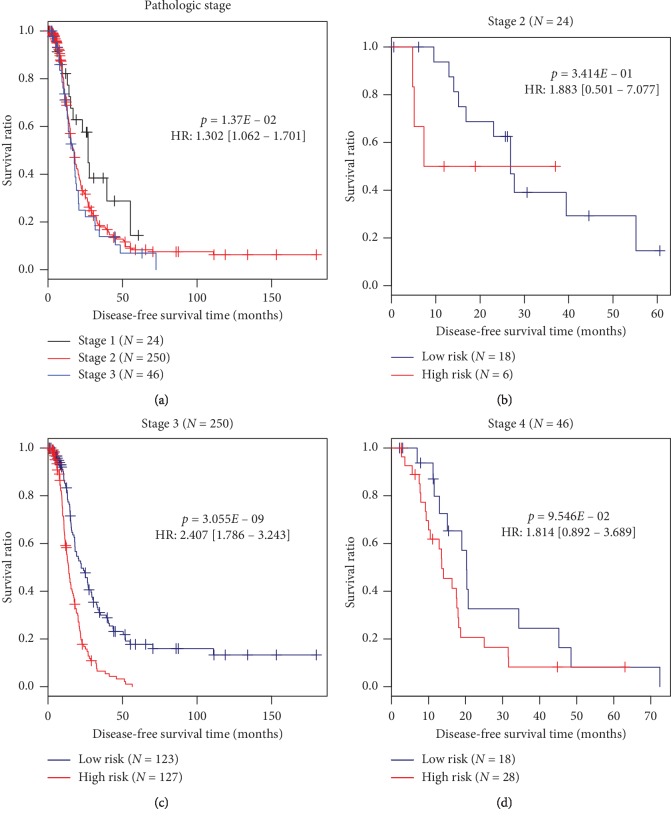
Kaplan–Meier survival curves for patients with different pathologic staging. (a) Prognostic value of pathologic stage; (b–d) prognostic value of multi-RNA-based risk score model stratified by pathologic staging ((b) stage 2; (c) stage 3; (d) stage 4). HR, hazard ratio.

Univariate and multivariate Cox regression analyses demonstrated that the prognostic value of this multi-RNA-based signature was independent of other clinical features in the training and entire datasets (Table 3). Furthermore, the pathologic stage was also found to be an independent prognostic factor for OvCa patients, with a higher stage having poor DFS (Table 3; Figure 4). To investigate whether our multi-RNA-based signature added additional prognostic values to the commonly used pathologic stage, stratification analyses were also conducted. The results showed this multi-RNA-based signature can further distinguish the survival of patients at the same stage 3 (Figure 4). The survival of patients with stages 2 and 4 not significantly separated by this multi-RNA-based signature may be related to small sample size in these subgroups.

**Figure 5:**
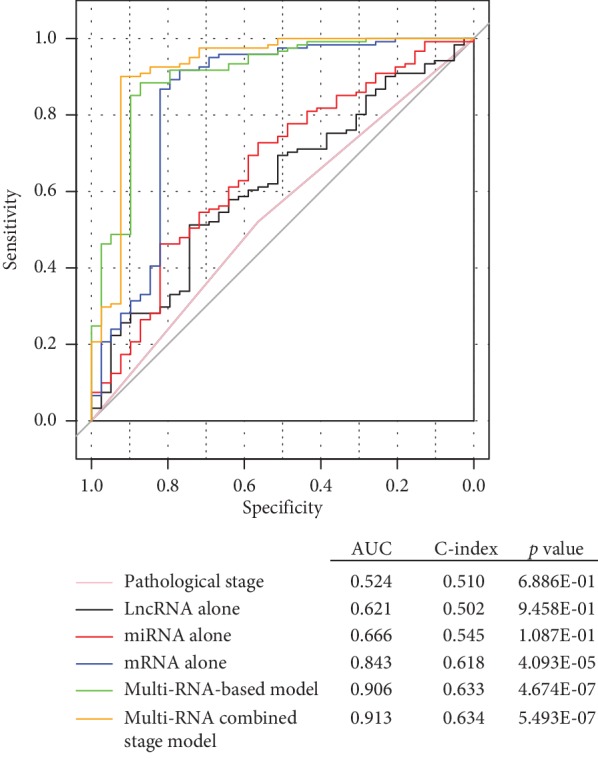
Comparison of the prognostic accuracy among the risk score model, pathologic staging, and mRNA, miRNA, and lncRNA alone. This analysis was performed using the training dataset. mRNA, messenger RNA; lncRNA, long noncoding RNA; miRNA, microRNA; ROC, receiver operator characteristic; AUC, area under curve; C-index, Harrell's concordance index. *p* value indicates the association between predicted survival and actually observed survival.

The superiority of the risk score model to clinical factors in survival prediction was also validated by comparison of AUC and C-index for each classifier. As anticipated, the results revealed that the AUC of the multi-RNA-based model was relatively higher than that of the pathologic stage and single RNA, but nearly similar to the combined model using the multi-RNA and pathologic stage (Figure 5). These findings implied that our multi-RNA based signature may be a promising biomarker for clinical use to predict the DFS of OvCa patients.

### 3.4. Functional Analysis for Prognostic Signature

To explore the underlying molecular mechanisms of these 14 RNAs in the signature, coexpression network between DELs and DEGs, ceRNA network among DELs, DEMs, and DEGs, and PPI networks between DEGs were constructed.

**Figure 6:**
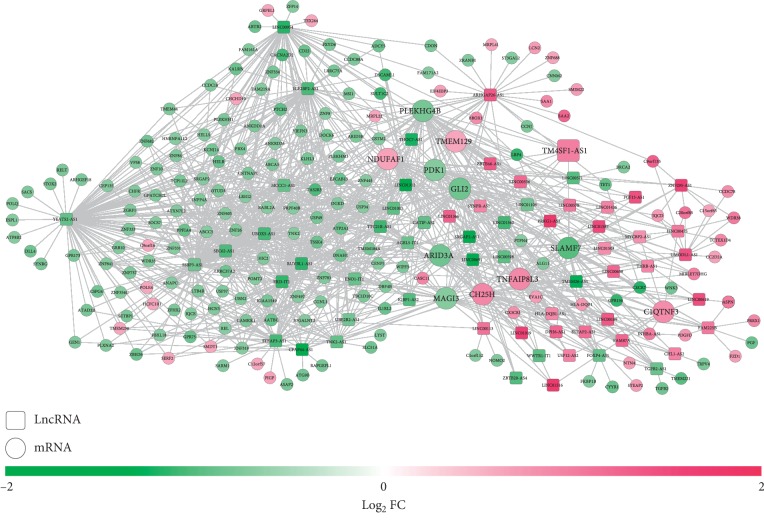
A coexpression network between differentially expressed lncRNAs and protein-coding mRNAs. Red, upregulated; green, downregulated. Circular, differentially expressed genes; square, lncRNAs. FC, fold change; mRNA, messenger RNA; lncRNA, long noncoding RNA. The genes with larger sizes were prognosis-related.

A total of 1086 coexpression pairs were identified between 67 DELs and 191 DEGs and were used to establish the coexpression network (Figure 6), in which 14 prognostic genes were also found to have positive coexpression with several lncRNAs, such as LINC00598-GLI2/ARID3A/PLEKHG4B/MAGI3/PDK1/SLAMF7, LINC00659-TMEM129, LINC00475-TNFAIP8L3/C1QTNF3/NDUFAF1, and LINC00189-CH25H (all PCC > 0.4). Function analysis showed that ARID3A was associated with Generic Transcription Pathway_Homo sapiens_R-HSA-212436; GLI2 was enriched in the Hedgehog signaling pathway, Basal cell carcinoma, and positive regulation of DNA replication (GO:0045740); and C1QTNF3 was enriched in regulation of cytokine secretion (GO:0050707) (Table 4).

**Figure 7:**
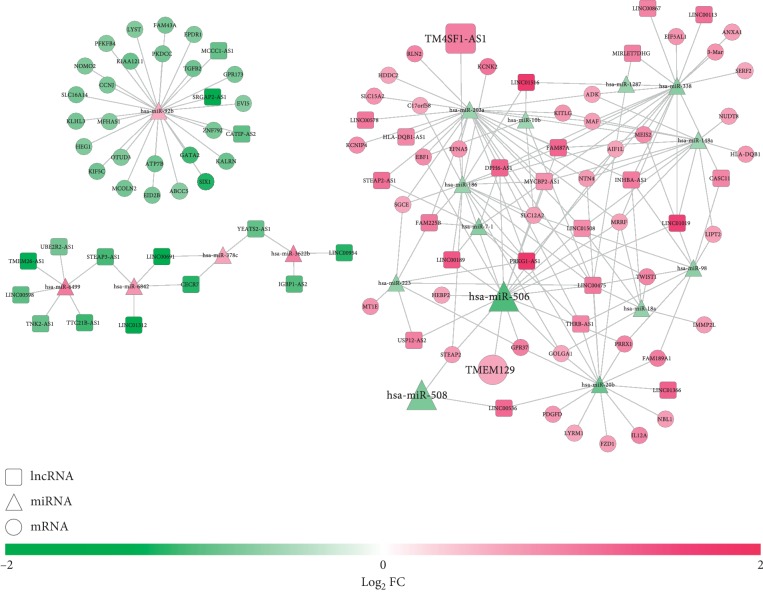
A competing endogenous RNA network among differentially expressed lncRNAs, miRNAs, and protein-coding mRNAs. Red, upregulated; green, downregulated. Circular, mRNAs; square, lncRNAs; triangle, miRNAs. FC, fold change; mRNA, messenger RNA; lncRNA, long noncoding RNA; miRNA, microRNA. The genes with larger sizes were prognosis-related.

A total of 38 DELs were predicted to interact with 18 DEMs by the DIANA-LncBase database, which formed 90 interaction pairs, while 13 DEMs were predicted to interact with 66 DEGs by the StarBase database to constitute 104 interaction pairs. These DEL-DEM and DEM-DEG interaction pairs were integrated to construct the ceRNA network (Figure 7), in which prognostic signature lncRNA TM4SF1-AS1 may function as a ceRNA for miR-186 to regulate STEAP2; prognostic signature miR-508 and miR-506 related ceRNA axes were LINC00536-miR-508-STEAP2 and LINC00475-miR-506-TMEM129, respectively. Function enrichment analysis showed that STEAP2 was involved in mineral absorption, transmembrane transport of small molecules_Homo sapiens_R-HSA-382551, copper ion import (GO:0015677), copper ion transport (GO:0006825), and iron ion import across plasma membrane (GO:0098711); TMEM129 was involved in ubiquitin-protein ligase activity involved in ERAD pathway (GO:1904264); PFKFB4 was enriched in positive regulation of glycolytic process (GO:0045821) and positive regulation of coenzyme metabolic process (GO:0051197) (Table 5).

**Figure 8:**
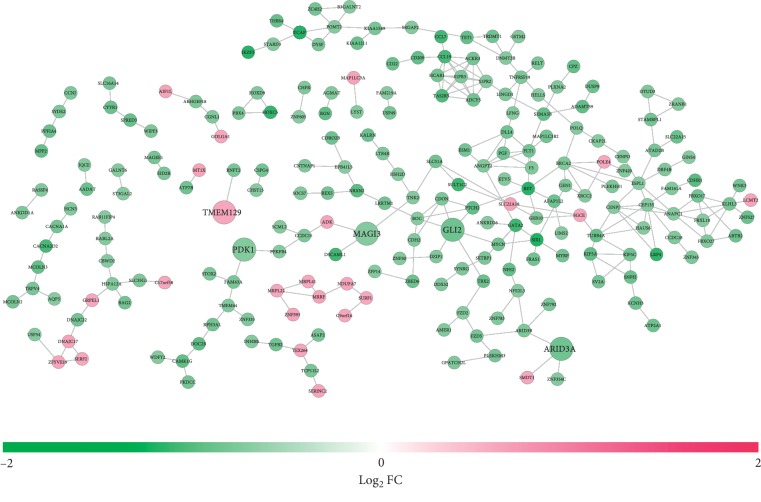
A protein-protein interaction network between differentially expressed protein-coding mRNAs. Red, upregulated; green, downregulated. mRNA, messenger RNA; FC, fold change. The genes with larger sizes were prognosis-related.

A total of 265 PPI pairs were predicted between 223 DEGs using the STRING database, which was used to construct the PPI network (Figure 8). In this network, prognostic GLI2, ARID3A, MAGI3, PDK1, and TMEM129 were included. Function enrichment analysis showed that GLI2 was involved in Hedgehog signaling pathway, Basal cell carcinoma, Hippo signaling pathway, pathways in cancer, and regulation of transcription, DNA-templated (GO:0006355); ARID3A was enriched in transcription regulatory region sequence-specific DNA binding (GO:0000976) and RNA polymerase II regulatory region sequence-specific DNA binding (GO:0000977); MAGI3 could interact with TNK2 to participate in positive regulation of protein phosphorylation (GO:0001934) and growth factor receptor binding (GO:0070851) (Table 6). Additionally, PDK1 could interact with PFKFB4 and thus may take part in the GO terms as described in the function enrichment results of the ceRNA network.

## 4. Discussion

In the present study, we, for the first time, constructed a multi-RNA-based signature (consisting of 11 mRNAs, 2 miRNAs, and 1 lncRNA) to predict the recurrence and DFS for OvCa patients. The high- and low-risk patients classified by the median risk score were shown to exhibit significantly different recurrence risks and DFS time. The AUC was 0.975, 0.912, and 0.901 for prediction of 1-, 3- and 5-year DFS in the training set, respectively, which seemed to be higher compared with other single RNA signatures reported previously, including Yang et al. (6-lncRNA, AUC = 0.813 at 3 year) [6], Bagnoli et al. (35-miRNA, AUC = 0.72) [23], and Zhao et al. screened (2-mRNA, AUC = 0.6075) [9]. This superiority of multi-RNA risk score to single RNA was also validated in our study according to the AUC (0.906 versus 0.621 for lncRNA, 0.666 for miRNAs, and 0.843 for mRNAs) and C-index results (0.633 versus 0.502 for lncRNA, 0.545 for miRNAs, and 0.618 for mRNAs) and was in line with the study of Xiong et al. [11].

Clinically, the pathologic staging is commonly considered as the gold standard for the assessment of the recurrence risk [24] and prognosis [25] of OvCa patients. This conclusion was also confirmed in our study, showing that pathologic staging was an independent factor for the prediction of DFS (that is, the prognosis was the worst in stage 4) after multivariate Cox regression analysis. However, some studies revealed that patients at the same pathological stage also had different recurrence risks and prognostic outcomes [26, 27]. Thus, there is a strong need to explore more effective prognostic biomarkers to independently or jointly identify the recurrence risk and prognosis of patients. Recently, several studies on other cancers had demonstrated that the risk score established by molecular signature had a similar or even higher accuracy for prognostic prediction than clinical features and the AUC (and/or C-index) was the largest for the combination of them [11, 28, 29]. Hereby, we also calculated the AUC (and/or C-index) of our multi-RNA-based risk score and independent pathologic stage factor. In accordance with previous studies [11, 28, 29], we also found that the AUC (and/or C-index) of our multi-RNA-based risk score was relatively higher than that of the pathologic stage, and the predicted and actually observed survival was significantly correlated using the risk score (*p*=4.674*E* − 07), but not pathologic stage (*p*=6.886*E* − 01). The stratification analysis also showed that the risk score can further distinguish the survival of patients at the same stage 3. These findings implied the insufficiency to use pathologic staging for prognosis prediction in the clinic and the advantage of our risk score. Of course, the optimum was still the combined model, with the AUC and C-index of 0.913 and 0.634, respectively.

There was no study to investigate the roles of lncRNA TM4SF1-AS1 and it may be a novel signature gene for cancer. However, we can indirectly speculate its functions by its interacted miR-186-STEAP2 axis. Similar to mRNAs, lncRNAs also harbor miRNA-response elements (MREs) and thus they can completely bind to miRNAs, leading to fewer miRNAs interacted with mRNAs, while it is well known that miRNAs can negatively regulate gene expression by interfering their translation or stability (ceRNA hypothesis). Accordingly, the upregulation of TM4SF1-AS1 identified in our recurrent samples may result in the overexpression of STEAP2 and possible lower expression of miR-186 in OvCa. This theory was validated in our study and previous studies: extensive evidence had suggested that miR-186 was a tumor suppressor gene, with a downregulated expression level in cancer tissues, cells [30, 31], and blood of recurrent oral squamous cell carcinoma patients compared with controls [32], including OvCa [33]. Overexpression of miR-186 into cancer cells significantly inhibited proliferation, invasion, metastasis [29, 34, 35], and induced mesenchymal-to-epithelial transition, G1 cell cycle arrest, and cell apoptosis [33]. STEAP2 (Seven Transmembrane Epithelial Antigen of the Prostate 2) is a gene primarily expressed in the prostate and the ovary is the only other area with a significant expression [36]. The roles of STEAP2 in OvCa may be similar to prostate cancer in which STEAP2 was observed to be upregulated and associated with advanced stage and Gleason score [37, 38]. The overexpression of STEAP2 induced the normal prostate cells to gain an ability to migrate and invade [36] while the knockdown of STEAP2 significantly reduced proliferation and migration of prostate cancer cells [39]. However, no study was performed to verify the interaction between miR-186-STEAP2 and TM4SF1-AS1, which may be the direction of our future search.

In addition to miR-186, we found that prognostic miR-508 also targeted STEAP2 and, hereby, miR-508 may be possibly downregulated in OvCa, which was also proved in our study and another literature [40]. Transfection of cancer cells with miR-508 mimics significantly suppressed cell proliferation, migration, and invasion while the use of miR-508 inhibitors resulted in an opposite trend [40, 41]. More interestingly, LINC00536 may be an upstream gene to influence the interaction between miR-508 and STEAP2 by acting as a ceRNA in the present study. Although their interaction was not reported, the opposed expression trend and roles of LINC00536 indirectly supported its potential relationship with miR-508. LINC00536 was identified to be highly expressed in bladder cancer tissues compared with controls and negatively associated with patients' survival rate. Function assays indicated that the knockdown of LINC00536 attenuated the malignant cell phenotypes *in vitro* and inhibited bladder cancer growth *in vivo* [42].

The present study showed that prognostic miR-506 was involved in OvCa by impacting the LINC00475-miR-506-TMEM129 ceRNA axis. This was also a novel mechanism explained for OvCa because no study reported their interactions previously, except that the expression level of each gene was preliminarily investigated as follows: Nam et al. revealed that the expression of miR-506-3p was significantly decreased in recurrent samples of OvCa patients compared with normal ovarian tissue and primary tumors [43]. The high level of miR-506 was positively associated with the early FIGO stage and longer survival in OvCa [44]. The introduction of miR-506 in OvCa cells can inhibit its proliferation and dissemination and promote senescence [44, 45]. Mo et al. used the lncRNA microarray to identify the high expression of LINC00475 in gastric cancer tissues compared with paired nontumor tissues [46]. The study of Hou et al. revealed that LINC00475 was associated with the overall survival of patients with clear cell renal cell carcinoma [47]. Silencing of LINC00475 suppressed cell proliferation, migration, and invasion in renal cell carcinoma [48] and glioma cells [49] *in vitro*. TMEM129 encodes an E3 ubiquitin ligase that was reported to mediate endoplasmic reticulum-associated HLA class I degradation [50] while tumors with downregulation of MHC class I conferred a significantly poorer prognosis in OvCa patients [51]. These findings suggested that TMEM129 may be upregulated in OvCa. In line with these studies, we also found that miR-506 was downregulated and LINC00475 and TMEM129 were upregulated in recurrent samples compared with nonrecurrent controls.

As for the prognostic genes not explored in the ceRNA network, most of them had been shown to be associated with the development and progression of OvCa or other cancers previously in the literature. For example, C1QTNF3 (C1q and TNF related 3) was found to be upregulated in bowel metastasis samples than primary OvCa [52]. ARID3A (AT-rich interactive domain 3A) positivity was implied to be correlated with longer DFS and cancer-specific survival in patients with residual rectal cancer [53]. MAGI3 (PDZ domain-containing protein membrane-associated guanylate kinase inverted 3) was identified to be downregulated in glioma samples. Overexpression of MAGI3 inhibited proliferation, migration, and cell cycle progression of glioma cells and decreased subcutaneous tumor growth in mice by inactivation of Wnt/*β*-catenin signaling pathway. High expression of MAGI3 was also significantly associated with excellent overall survival [54]. TNFAIP8L3 (TNF alpha-induced protein 8 like 3, also known as TIPE3), a transfer protein of phosphoinositide second messengers, was detected to be significantly upregulated in breast cancer tissues (especially invasive or metastasized type) as compared with adjacent nontumor tissues. Inhibition of TNFAIP8L3 may block cancer cell proliferation, migration, and invasion *in vitro* and *in vivo* by inactivation of the AKT and NF-*κ*B signaling pathways [55, 56]. In consistence with these studies, we also found that C1QTNF3 and TNFAIP8L3 were upregulated, while ARID3A and MAGI3 were downregulated in recurrent samples, and they were respectively an oncogenic or tumor suppressor gene for prediction of DFS. Although there were also studies to confirm the roles of Gli2 (Glioma-associated oncogene family member 2) [57], PDK1 (pyruvate dehydrogenase kinase) [58], NDUFAF1 (NADH dehydrogenase 1 alpha subcomplex assembly factor 1) [59], SLAMF7 (SLAM family member 7) [60], and CH25H (cholesterol 25-hydroxylase) [61], their conclusions seemed to be opposite with our study, which may be attributed to the differences in cancer type or sample size and thus further validation experiments are needed. PLEKHG4B was not investigated in any study of cancer, but we speculated that its expression may be positively regulated by LINC00598 because of their PCC of 0.43. Knockdown of LINC00598 was previously proved to induce G0/G1 cell cycle arrest and inhibit proliferation, indicating its tumor-promoting roles [62]. Accordingly, we speculated that PLEKHG4B was also highly expressed in OvCa, which was verified in our study as expected.

## 5. Conclusion

In the present study, we used the recurrence-associated genes to construct a multi-RNA-based risk score model which was shown to effectively stratify OvCa patients into groups at low risk and high risk of shorter DFS. This model was not only independent of clinical features but also superior to commonly used pathologic staging in the clinic for prognostic prediction. Furthermore, we predicted several new interaction axes to explain the possible mechanisms of these RNAs in OvCa, such as TM4SF1-AS1-miR-186-STEAP2, LINC00536-miR-508-STEAP2, LINC00475-miR-506-TMEM129, and LINC00598-PLEKHG4B. However, some limitations still exist. First, quantitative PCR experiments should be performed to validate the expression and prognosis accuracy of our signature genes in clinical samples because there may be potential differences with TCGA sequencing data results as reported for Gli2, PDK1, NDUFAF1, SLAMF7, and CH25H. Second, *in vitro* and *in vivo* experiments are also essential to validate the ceRNA or coexpression mechanisms we identified.

## Figures and Tables

**Table 1:** The clinical features of ovarian cancer patients.

Clinical characteristics	Training set (*N* = 161)	Validation set (*N* = 161)	Entire set (*N* = 322)
Age (years, mean ± SD)	58.32 ± 11.38	59.17 ± 11.56	58.75 ± 11.46
Neoplasm histologic grade (G2/G3/-)	20/136/5	22/136/3	42/272/8
Pathologic stage (II/III/IV/-)	11/125/24/1	13/125/22/1	24/250/46/2
Lymphovascular invasion (yes/no/-)	41/24/96	43/20/98	84/44/194
Vascular invasion (yes/no/-)	26/21/114	30/16/115	56/37/229
Tumor recurrence (yes/no)	121/40	121/40	242/80
Disease-free survival time (months, mean ± SD)	21.67 ± 23.76	20.42 ± 19.89	21.04 ± 21.89

SD: standard deviation.

**Table 2:** The most optimal signature combination.

Type	Symbol	Expression		Univariate Cox regression analysis			LASSO coefficient
		logFC	FDR	HR	95% CI	*p* value	
miRNA	Hsa-miR-506	−1.01	3.91*E* − 02	0.94	0.88–0.99	0.014	−0.20
Coding	MAGI3	−0.71	1.15*E* − 02	0.56	0.41–0.78	0.001	−0.27
Coding	SLAMF7	−0.99	4.43*E* − 02	0.75	0.62–0.92	0.005	−0.29
Coding	GLI2	−0.85	3.58*E* − 02	0.74	0.59–0.93	0.010	−0.12
Coding	PDK1	−0.72	3.93*E* − 02	0.66	0.47–0.91	0.011	−0.08
Coding	ARID3A	−0.84	2.99*E* − 02	0.72	0.55–0.94	0.017	−0.02
Coding	PLEKHG4B	−0.79	1.25*E* − 02	0.80	0.65–0.97	0.027	−0.03
miRNA	Hsa-miR-508	−0.76	4.84*E* − 02	1.32	1.02–1.75	0.048	0.12
lncRNA	TM4SF1-AS1	0.99	2.88*E* − 03	1.28	1.01–1.62	0.037	0.04
Coding	TNFAIP8L3	0.89	6.30*E* − 03	2.082	1.37–3.16	0.001	0.29
Coding	C1QTNF3	0.85	1.70*E* − 02	1.331	1.11–1.59	0.002	0.13
Coding	NDUFAF1	0.65	2.24*E* − 03	1.791	1.22–2.62	0.003	0.15
Coding	CH25H	0.90	4.70*E* − 02	1.39	1.05–1.83	0.019	0.16
Coding	TMEM129	0.60	1.41*E* − 02	1.410	1.04–1.92	0.028	0.14

LASSO, least absolute shrinkage and selection operator; FC, fold change; FDR, false discovery rate; HR, hazard ratio; CI, confidence interval.

**Table 3:** Univariate and multivariable Cox regression analyses with clinical features.

Variables	Univariate analysis			Multivariate analysis		
	HR	95% CI	*p* value	HR	95% CI	*p* value
*Training set (N* *=* *161)*						
Age (years, mean ± SD)	1.015	0.997–1.032	9.56*E* − 02	—	—	—
Neoplasm histologic grade (G2/G3/-)	1.639	0.950–2.828	7.27*E* − 02	—	—	—
Pathologic stage (II/III/IV/-)	1.713	1.172–2.505	**5.68*E* − 03**	1.473	1.189–2.195	**4.68*E* − 02**
Lymphovascular invasion (yes/no/-)	1.345	0.693–2.610	3.74*E* − 01	—	—	—
Vascular invasion (yes/no/-)	1.321	0.614–2.835	4.76*E* − 01	—	—	—
Risk score status (high/low)	2.905	1.972–4.281	**2.12*E* − 08**	2.679	1.809–3.966	**8.61*E* − 07**
*Testing set (N* *=* *161)*						
Age (years, mean ± SD)	1.007	0.982–1.014	8.00*E* − 01	—	—	—
Neoplasm histologic grade (G2/G3/-)	1.107	0.487–1.337	4.04*E* − 01	—	—	—
Pathologic stage (II/III/IV/-)	1.198	0.662–1.451	9.20*E* − 01	—	—	—
Lymphovascular invasion (yes/no/-)	1.191	0.464–1.712	7.29*E* − 01	—	—	—
Vascular invasion (yes/no/-)	1.045	0.451–1.982	8.83*E* − 01	—	—	—
PS status (high/low)	1.891	1.315–2.721	**4.85*E* − 04**	—	—	—
*Entire set (N* *=* *322)*						
Age (years, mean ± SD)	1.006	0.994–1.017	3.41*E* − 01	—	—	—
Neoplasm histologic grade (G2/G3/-)	1.171	0.809–1.691	4.04*E* − 01	—	—	—
Pathologic stage (II/III/IV/-)	1.302	1.062–1.701	**1.37*E* − 02**	1.114	1.045–1.470	**4.43*E* − 02**
Lymphovascular invasion (yes/no/-)	1.093	0.687–1.737	7.08*E* − 01	—	—	—
Vascular invasion (yes/no/-)	1.125	0.661–1.914	6.64*E* − 01	—	—	—
Risk score status (high/low)	2.323	1.786–3.022	**1.16*E* − 10**	2.259	1.728–2.953	**2.49*E* − 09**

HR, hazard ratio; CI, confidence interval; SD, standard deviation. Bold indicates significance.

**Table 4:** Function enrichment analysis for the genes in the coexpression network.

	Term	*p* value	Genes
KEGG	Herpes simplex virus 1 infection	2.14*E* − 05	ZNF331; ZNF682; ZNF10; ZNF8; ZNF26; ZFP14; ZNF605; ZNF84; ZNF737; ZNF688; ZNF841; ZNF334; ZNF333; ZNF354 C; ZNF783; HLA-DQB1
	Hedgehog signaling pathway	1.02*E* − 02	PTCH2; GLI2; CDON
	Mannose type O-glycan biosynthesis	2.01*E* − 02	B3GALNT2; POMT2
	Basal cell carcinoma	2.24*E* − 02	FZD1; PTCH2; GLI2
Reactome	GLI proteins bind promoters of Hh responsive genes to promote transcription_Homo sapiens_R-HSA-5635851	1.85*E* − 03	PTCH2; GLI2
	Signaling by Hedgehog_Homo sapiens_R-HSA-5358351	1.98*E* − 03	PTCH2; IQCE; WDR35; CDON; GLI2; ADCY5
	Hedgehog 'on' state_Homo sapiens_R-HSA-5632684	8.95*E* − 03	PTCH2; IQCE; GLI2; CDON
	Cardiac conduction_Homo sapiens_R-HSA-5576891	9.63*E* − 03	KCNJ14; FKBP1B; CACNA2D2; FXYD6; ATP2A1
	Activation of SMO_Homo sapiens_R-HSA-5635838	1.26*E* − 02	IQCE; CDON
	Ion homeostasis_Homo sapiens_R-HSA-5578775	1.28*E* − 02	FKBP1B; ATP2A1; FXYD6
	Ion channel transport_Homo sapiens_R-HSA-983712	1.35*E* − 02	ATP8B2; TRPV4; WNK3; FKBP1B; FXYD6; ATP2A1
	ECM proteoglycans_Homo sapiens_R-HSA-3000178	1.56*E* − 02	TGFB2; LRP4; ASPN
	Ion transport by P-type ATPases_Homo sapiens_R-HSA-936837	1.56*E* − 02	ATP8B2; ATP2A1; FXYD6
	Generic Transcription Pathway_Homo sapiens_R-HSA-212436	2.42*E* − 02	ZNF331; ZNF682; ZNF10; ARID3A; ZNF26; ZFP14; ZNF605; CENPJ; ZNF737; ZNF688; ZNF445; ZNF334; ZNF333; ZNF354 C
	Adrenaline, noradrenaline inhibits insulin secretion_Homo sapiens_R-HSA-400042	2.91*E* − 02	CACNA2D2; ADCY5
	Resolution of D-loop Structures through Holliday Junction Intermediates_Homo sapiens_R-HSA-5693568	3.73*E* − 02	GEN1; BRCA2
	Resolution of D-Loop Structures_Homo sapiens_R-HSA-5693537	3.95*E* − 02	GEN1; BRCA2
	Muscle contraction_Homo sapiens_R-HSA-397014	4.02*E* − 02	KCNJ14; FKBP1B; CACNA2D2; FXYD6; ATP2A1
	IRS activation_Homo sapiens_R-HSA-74713	4.69*E* − 02	GRB10
	Hyaluronan biosynthesis and export_Homo sapiens_R-HSA-2142850	4.69*E* − 02	ABCC5
	Leukotriene receptors_Homo sapiens_R-HSA-391906	4.69*E* − 02	LTB4R
	Scavenging by Class B Receptors_Homo sapiens_R-HSA-3000471	4.69*E* − 02	SAA1
GO BP	Spinal cord dorsal/ventral patterning (GO:0021513)	1.33*E* − 03	DLL4; GLI2
	Regulation of DNA replication (GO:0006275)	2.92*E* − 03	CCDC88A; USP37; DBF4B; GLI2
	Central nervous system projection neuron axonogenesis (GO:0021952)	3.88*E* − 03	C12ORF57; GLI2
	Embryonic digestive tract development (GO:0048566)	1.54*E* − 02	TGFB2; GLI2
	Dorsal/ventral pattern formation (GO:0009953)	1.54*E* − 02	DSCAML1; GLI2
	Renal system development (GO:0072001)	1.97*E* − 02	TGFB2; LRP4; GLI2
	Kidney development (GO:0001822)	2.33*E* − 02	TGFB2; LRP4; GLI2
	Smoothened signaling pathway (GO:0007224)	3.52*E* − 02	CC2D2A; GLI2
	Regulation of cytokine secretion (GO:0050707)	3.73*E* − 02	C1QTNF3; SAA1
	Positive regulation of protein secretion (GO:0050714)	4.52*E* − 02	C1QTNF3; TGFB2; SAA1
	Positive regulation of DNA replication (GO:0045740)	4.62*E* − 02	DBF4B; GLI2
GO MF	Ubiquitin-like protein-specific protease activity (GO:0019783)	7.57*E* − 04	USP37; ZRANB1; USP49; USP34; OTUD3
	Thiol-dependent ubiquitin-specific protease activity (GO:0004843)	8.54*E* − 04	USP37; ZRANB1; USP49; USP34; OTUD3
	Thiol-dependent ubiquitinyl hydrolase activity (GO:0036459)	1.99*E* − 03	USP37; ZRANB1; USP49; USP34; OTUD3
	Oxidoreductase activity, acting on paired donors, with incorporation or reduction of molecular oxygen, 2-oxoglutarate as one donor, and incorporation of one atom each of oxygen into both donors (GO:0016706)	3.73*E* − 02	BBOX1; TET1
	Transition metal ion binding (GO:0046914)	3.87*E* − 02	ZNF331; ZNF593; ZNF84; LCN2; TET1; BBOX1; ZNF8; GLI2

KEGG, Kyoto Encyclopedia of Genes and Genomes; GO, Gene Ontology; BP, biological process; MF, molecular function.

**Table 5:** Function enrichment analysis for genes in the ceRNA network.

	Term	*p* value	Genes
KEGG	Inflammatory bowel disease (IBD)	6.29*E* − 05	TGFB2; MAF; IL12A; HLA-DQB1
	Leishmaniasis	1.88*E* − 03	TGFB2; IL12A; HLA-DQB1
	Th1 and Th2 cell differentiation	3.50*E* − 03	MAF; IL12A; HLA-DQB1
	Toxoplasmosis	6.21*E* − 03	TGFB2; IL12A; HLA-DQB1
	Allograft rejection	6.98*E* − 03	IL12A; HLA-DQB1
	Type I diabetes mellitus	8.88*E* − 03	IL12A; HLA-DQB1
	Malaria	1.14*E* − 02	TGFB2; IL12A
	Mineral absorption	1.23*E* − 02	STEAP2; MT1E
	MAPK signaling pathway	1.63*E* − 02	TGFB2; KITLG; PDGFD; EFNA5
	Tuberculosis	2.14*E* − 02	TGFB2; IL12A; HLA-DQB1
	Proteoglycans in cancer	2.88*E* − 02	FZD1; TGFB2; TWIST1
	Rap1 signaling pathway	3.07*E* − 02	KITLG; PDGFD; EFNA5
	TGF-beta signaling pathway	3.57*E* − 02	TGFB2; NBL1
	Rheumatoid arthritis	3.64*E* − 02	TGFB2; HLA-DQB1
	Amoebiasis	4.01*E* − 02	TGFB2; IL12A
	Hematopoietic cell lineage	4.09*E* − 02	KITLG; HLA-DQB1
	Ras signaling pathway	4.13*E* − 02	KITLG; PDGFD; EFNA5
	Melanogenesis	4.39*E* − 02	FZD1; KITLG
	Chagas disease (American trypanosomiasis)	4.55*E* − 02	TGFB2; IL12A
BioCarta	Wnt/LRP6 Signalling_Homo sapiens_h_wnt-lrp6Pathway	2.29*E* − 02	FZD1
	Role of Parkin in Ubiquitin-Proteasomal Pathway_Homo sapiens_h_parkinPathway	2.61*E* − 02	GPR37
	NO2-dependent IL 12 Pathway in NK cells_Homo sapiens_h_no2il12Pathway	2.93*E* − 02	IL12A
	Melanocyte Development and Pigmentation Pathway_Homo sapiens_h_melanocytepathway	4.21*E* − 02	KITLG
	IL12 and Stat4 Dependent Signaling Pathway in Th1 Development_Homo sapiens_h_IL12Pathway	4.84*E* − 02	IL12A
Reactome	Transmembrane transport of small molecules_Homo sapiens_R-HSA-382551	1.35*E* − 02	SLC12A2; ATP7B; SLC15A2; ABCC5; STEAP2; MCOLN2
	Hyaluronan biosynthesis and export_Homo sapiens_R-HSA-2142850	1.64*E* − 02	ABCC5
	Cation-coupled Chloride cotransporters_Homo sapiens_R-HSA-426117	2.29*E* − 02	SLC12A2
	Peptide ligand-binding receptors_Homo sapiens_R-HSA-375276	2.60*E* − 02	GPR37; ANXA1; RLN2
	Formyl peptide receptors bind formyl peptides and many other ligands_Homo sapiens_R-HSA-444473	2.61*E* − 02	ANXA1
	Relaxin receptors_Homo sapiens_R-HSA-444821	2.61*E* − 02	RLN2
	Muscle contraction_Homo sapiens_R-HSA-397014	2.70*E* − 02	ANXA1; KCNIP4; KCNK2
	Response to metal ions_Homo sapiens_R-HSA-5660526	3.57*E* − 02	MT1E
	Metallothioneins bind metals_Homo sapiens_R-HSA-5661231	3.57*E* − 02	MT1E
	Tandem pore domain potassium channels_Homo sapiens_R-HSA-1296346	3.89*E* − 02	KCNK2
	EPH-Ephrin signaling_Homo sapiens_R-HSA-2682334	3.93*E* − 02	EFNA5; KALRN
	Purine salvage_Homo sapiens_R-HSA-74217	4.21*E* − 02	ADK
	Transport of inorganic cations/anions and amino acids/oligopeptides_Homo sapiens_R-HSA-425393	4.24*E* − 02	SLC12A2; SLC15A2
	MHC class II antigen presentation_Homo sapiens_R-HSA-2132295	4.55*E* − 02	KIF5C; HLA-DQB1
GO BP	Copper ion import (GO:0015677)	2.96*E* − 04	ATP7B; STEAP2
	Digestive tract development (GO:0048565)	4.13*E* − 04	TGFB2; PKDCC; GATA2
	Embryonic organ development (GO:0048568)	7.55*E* − 04	TGFB2; KITLG; PKDCC
	Copper ion transport (GO:0006825)	9.51*E* − 04	ATP7B; STEAP2
	Embryonic cranial skeleton morphogenesis (GO:0048701)	1.10*E* − 03	SIX1; TWIST1
	Embryonic digestive tract development (GO:0048566)	1.96*E* − 03	TGFB2; PKDCC
	Embryonic skeletal system morphogenesis (GO:0048704)	2.37*E* − 03	SIX1; TWIST1
	Regulation of phosphatidylinositol 3-kinase signaling (GO:0014066)	2.44*E* − 03	TGFB2; PDGFD; TWIST1
	Regulation of angiogenesis (GO:0045765)	2.85*E* − 03	TGFB2; RLN2; TWIST1; GATA2
	Iron ion import across plasma membrane (GO:0098711)	1.96*E* − 02	STEAP2
	Carbohydrate phosphorylation (GO:0046835)	3.57*E* − 02	ADK
	Muscle cell migration (GO:0014812)	3.57*E* − 02	SIX1
	Ribonucleoside monophosphate biosynthetic process (GO:0009156)	4.52*E* − 02	ADK
	Purine-containing compound salvage (GO:0043101)	4.52*E* − 02	ADK
	Positive regulation of glycolytic process (GO:0045821)	4.84*E* − 02	PFKFB4
	Positive regulation of coenzyme metabolic process (GO:0051197)	4.84*E* − 02	PFKFB4
	Positive regulation of cellular catabolic process (GO:0031331)	4.87*E* − 02	PFKFB4; TWIST1
GO MF	Transcriptional activator activity, RNA polymerase II transcription regulatory region sequence-specific binding (GO:0001228)	1.46*E* − 02	EBF1; SIX1; GATA2; MEIS2
	Neurotrophin TRKA receptor binding (GO:0005168)	1.96*E* − 02	EFNA5
	Transcriptional activator activity, RNA polymerase II core promoter proximal region sequence-specific binding (GO:0001077)	2.05*E* − 02	EBF1; SIX1; MEIS2
	NAADP-sensitive calcium-release channel activity (GO:0072345)	2.29*E* − 02	MCOLN2
	Type II transforming growth factor beta receptor binding (GO:0005114)	2.61*E* − 02	TGFB2
	Phosphofructokinase activity (GO:0008443)	2.61*E* − 02	PFKFB4
	Carbohydrate kinase activity (GO:0019200)	4.21*E* − 02	ADK
	Ubiquitin protein ligase activity involved in ERAD pathway (GO:1904264)	4.21*E* − 02	TMEM129
	Platelet-derived growth factor receptor binding (GO:0005161)	4.21*E* − 02	PDGFD
	Nucleoside kinase activity (GO:0019206)	4.52*E* − 02	ADK

KEGG, Kyoto Encyclopedia of Genes and Genomes; GO, Gene Ontology; BP, biological process; MF, molecular function.

**Table 6:** Function enrichment analysis for genes in the PPI network.

	Term	*p* value	Genes
KEGG	Hedgehog signaling pathway	1.84*E* − 03	BOC; PTCH2; GLI2; CDON
	Basal cell carcinoma	5.36*E* − 03	FZD2; FZD5; PTCH2; GLI2
	Other types of O-glycan biosynthesis	2.47*E* − 02	POMT2; LFNG
	Mannose type O-glycan biosynthesis	2.68*E* − 02	B3GALNT2; POMT2
	Hippo signaling pathway	3.38*E* − 02	TGFB2; FZD2; FZD5; RASSF4; GLI2
	Pathways in cancer	3.60*E* − 02	RET; DLL4; GSTM2; TGFB2; FZD2; FZD5; PTCH2; BRCA2; PGF; GLI2; ADCY5
	Mucin type O-glycan biosynthesis	4.66*E* − 02	GALNT6; ST3GAL2
	Cytokine-cytokine receptor interaction	4.72*E* − 02	TGFB2; CCL7; TNFRSF19; ACKR3; INHBB; CCL19; RELT
	MAPK signaling pathway	4.80*E* − 02	TGFB2; FLT1; ANGPT2; CACNA2D2; CACNA1A; DUSP9; PGF
BioCarta	Rho cell motility signaling pathway_Homo sapiens_h_rhoPathway	4.93*E* − 02	CACNA1A; ASAP2
Reactome	GLI proteins bind promoters of Hh responsive genes to promote transcription_Homo sapiens_R-HSA-5635851	4.63*E* − 05	BOC; PTCH2; GLI2
	Hedgehog 'on' state_Homo sapiens_R-HSA-5632684	3.77*E* − 04	BOC; PTCH2; IQCE; DZIP1; GLI2; CDON
	Signaling by Hedgehog_Homo sapiens_R-HSA-5358351	8.44*E* − 04	PTCH2; BOC; IQCE; DZIP1; CDON; GLI2; ADCY5
	Signal Transduction_Homo sapiens_R-HSA-162582	1.50*E* − 02	RET; AMER1; FLT1; WIPF3; USP34; ATP2A1; KALRN; LTB4R; THBS4; GLI2; ADCY5; LFNG; SYDE2; SPRED3; DLL4; HCAR1; CCL7; KIF5A; BOC; GRB10; S1PR3; CCL19; S1PR2; SRGAP2; TAS2R5; CDON; PDK1; LINGO1; FZD2; FZD5; PTCH2; IQCE; ARHGEF18; INHBB; DUSP9; PGF; ACKR3; CENPO; DZIP1
GO BP	Embryonic digestive tract development (GO:0048566)	1.36*E* − 03	TGFB2; PKDCC; GLI2
	Digestive tract development (GO:0048565)	1.44*E* − 03	TGFB2; PKDCC; GATA2; GLI2
	Spinal cord dorsal/ventral patterning (GO:0021513)	1.80*E* − 03	DLL4; GLI2
	Renal system development (GO:0072001)	4.50*E* − 03	TGFB2; SIX1; LRP4; GLI2
	Kidney development (GO:0001822)	5.67*E* − 03	TGFB2; SIX1; LRP4; GLI2
	Axon guidance (GO:0007411)	8.94*E* − 03	RET; KIF5C; KIF5A; GRB10; PLXNA2; GLI2
	Odontogenesis (GO:0042476)	1.14*E* − 02	TGFB2; AQP5; GLI2
	Branching morphogenesis of an epithelial tube (GO:0048754)	1.29*E* − 02	DLL4; SIX1; GLI2
	Axonogenesis (GO:0007409)	1.31*E* − 02	RET; LINGO1; KIF5C; KIF5A; GRB10; DSCAML1; GLI2
	Dorsal/ventral pattern formation (GO:0009953)	2.06*E* − 02	DSCAML1; GLI2
	Embryonic skeletal system development (GO:0048706)	2.26*E* − 02	SIX1; DSCAML1
	Cell morphogenesis involved in neuron differentiation (GO:0048667)	2.41*E* − 02	LINGO1; CDHR1; LRP4; DSCAML1
	Embryonic skeletal system morphogenesis (GO:0048704)	2.47*E* − 02	SIX1; DSCAML1
	Positive regulation of nucleic acid-templated transcription (GO:1903508)	2.60*E* − 02	RET; NFE2; AFAP1L2; FZD2; MYCN; MYRF; NFE2L3; SIX1; PBX4; BRCA2; GLI2
	Heart development (GO:0007507)	2.66*E* − 02	TGFB2; TCAP; GATA2; GLI2; TBX2
	Endocrine system development (GO:0035270)	3.62*E* − 02	SIX1; GLI2
	Neuron projection morphogenesis (GO:0048812)	3.69*E* − 02	LINGO1; CNTNAP1; LRP4; SRGAP2; DSCAML1
	Skeletal system morphogenesis (GO:0048705)	4.93*E* − 02	SIX1; DSCAML1
GO MF	Transcription regulatory region sequence-specific DNA binding (GO:0000976)	5.77*E* − 03	NFE2; CUX1; NFE2L3; ARID3A; ARID3B; GATA2; ZBED6; HOXD9; ETV5
	RNA polymerase II regulatory region DNA binding (GO:0001012)	7.67*E* − 03	CUX1; ARID3A; ARID3B; GATA2; ZBED6; HOXD9; ETV5
	Guanylate kinase activity (GO:0004385)	8.90*E* − 03	MPP2; MAGI3
	Growth factor receptor binding (GO:0070851)	2.03*E* − 02	LINGO1; ESM1; TNK2; PGF
	Protein tyrosine kinase activity (GO:0004713)	2.53*E* − 02	RET; PKDCC; FLT1; CUX1; TNK2
	Nucleotide kinase activity (GO:0019201)	2.68*E* − 02	MPP2; MAGI3
	Epidermal growth factor receptor binding (GO:0005154)	3.37*E* − 02	LINGO1; TNK2
	RNA polymerase II regulatory region sequence-specific DNA binding (GO:0000977)	3.45*E* − 02	MYCN; CUX1; SIX1; ARID3A; ARID3B; GATA2; ZBED6; HOXD9; ETV5; TBX2
	WW domain binding (GO:0050699)	4.39*E* − 02	NFE2; TNK2

PPI, protein-protein interaction; KEGG, Kyoto Encyclopedia of Genes and Genomes; GO, Gene Ontology; BP, biological process; MF, molecular function.

## Data Availability

All data can be available in TCGA database (https://portal.gdc.cancer.gov/).

## Supplementary Materials

Supplementary Materials Supplemental Information 1: risk score calculated by each patient in three datasets.

## References

[1] Siegel R. L., Miller K. D., Jemal A. (2018). Cancer statistics, 2018. *CA: A Cancer Journal for Clinicians*.

[2] Chen W., Zheng R., Baade P. D. (2016). Cancer statistics in China, 2015. *CA: A Cancer Journal for Clinicians*.

[3] Edwards M. K., Noer M. C., Sperling C. D. (2007). Survival of ovarian cancer patients in Denmark: results from the Danish gynaecological cancer group (DGCG) database, 1995–2012. *Acta Oncologica*.

[4] Timmermans M., Sonke G. S., Van de Vijver K. K., van der Aa M. A., Kruitwagen R. F. P. M. (2018). No improvement in long-term survival for epithelial ovarian cancer patients: a population-based study between 1989 and 2014 in The Netherlands. *European Journal of Cancer*.

[5] Vistad I., Bjørge L., Solheim O. (2017). A national, prospective observational study of first recurrence after primary treatment for gynecological cancer in Norway. *Acta Obstetricia et Gynecologica Scandinavica*.

[6] Yang K., Hou Y., Li A. (2017). Identification of a six-lncRNA signature associated with recurrence of ovarian cancer. *Scientific Reports*.

[7] Dong J., Xu M. (2019). A 19-miRNA Support Vector Machine classifier and a 6-miRNA risk score system designed for ovarian cancer patients. *Oncology Reports*.

[8] Zhou J., Li L., Wang L., Li X., Xing H., Cheng L. (2018). Establishment of a SVM classifier to predict recurrence of ovarian cancer. *Molecular Medicine Reports*.

[9] Zhao H., Guo E., Hu T. (2016). KCNN4 and S100A14 act as predictors of recurrence in optimally debulked patients with serous ovarian cancer. *Oncotarget*.

[10] Shukla S., Evans J. R., Malik R. (2017). Development of a RNA-seq based prognostic signature in lung adenocarcinoma. *Journal of the National Cancer Institute*.

[11] Xiong Y., Wang R., Peng L. (2017). An integrated lncRNA, microRNA and mRNA signature to improve prognosis prediction of colorectal cancer. *Oncotarget*.

[12] Wang X., Han L., Zhou L., Wang L., Zhang L. M. (2018). Prediction of candidate RNA signatures for recurrent ovarian cancer prognosis by the construction of an integrated competing endogenous RNA network. *Oncology Reports*.

[13] Povey S., Lovering R., Bruford E., Wright M., Lush M., Wain H. (2001). The HUGO gene Nomenclature committee (HGNC). *Human Genetics*.

[14] Ritchie M. E., Phipson B., Wu D. (2015). Limma powers differential expression analyses for RNA-sequencing and microarray studies. *Nucleic Acids Research*.

[15] Goeman J. J. (2010). L1 penalized estimation in the Cox proportional hazards model. *Biometrical Journal*.

[16] Tibshirani R. (1997). The lasso method for variable selection in the Cox model. *Statistics in Medicine*.

[17] Schröder M. S., Culhane A. C., Quackenbush J., Haibe-Kains B. (2011). survcomp: an R/Bioconductor package for performance assessment and comparison of survival models. *Bioinformatics*.

[18] Paraskevopoulou M. D., Georgakilas G., Kostoulas N. (2013). DIANA-LncBase: experimentally verified and computationally predicted microRNA targets on long non-coding RNAs. *Nucleic Acids Research*.

[19] Yang J.-H., Li J.-H., Shao P., Zhou H., Chen Y.-Q., Qu L.-H. (2011). starBase: a database for exploring microRNA-mRNA interaction maps from Argonaute CLIP-Seq and Degradome-Seq data. *Nucleic Acids Research*.

[20] Xu J., Zhang J., Shan F., Wen J., Wang Y. (2019). SSTR5-AS1 functions as a ceRNA to regulate CA2 by sponging miR-15b-5p for the development and prognosis of HBV-related hepatocellular carcinoma. *Molecular Medicine Reports*.

[21] Szklarczyk D., Franceschini A., Wyder S. (2015). STRING v10: protein-protein interaction networks, integrated over the tree of life. *Nucleic Acids Research*.

[22] Kuleshov M. V., Jones M. R., Rouillard A. D. (2016). Enrichr: a comprehensive gene set enrichment analysis web server 2016 update. *Nucleic Acids Research*.

[23] Bagnoli M., Canevari S., Califano D. (2016). Development and validation of a microRNA-based signature (MiROvaR) to predict early relapse or progression of epithelial ovarian cancer: a cohort study. *The Lancet Oncology*.

[24] Chang C., Chen J., Chen W.-A., Ho S.-P., Liou W. S., Chiang A. J. (2016). Assessing the risk of clinical and pathologic factors for relapse of borderline ovarian tumours. *Journal of Obstetrics and Gynaecology*.

[25] Kempf E., Desamericq G., Vieites B. (2017). Clinical and pathologic features of patients with non-epithelial ovarian cancer: retrospective analysis of a single institution 15-year experience. *Clinical and Translational Oncology*.

[26] Baek S.-J., Park J.-Y., Kim D.-Y. (2008). Stage IIIC epithelial ovarian cancer classified solely by lymph node metastasis has a more favorable prognosis than other types of stage IIIC epithelial ovarian cancer. *Journal of Gynecologic Oncology*.

[27] Gouy S., Saidani M., Maulard A. (2018). Characteristics and prognosis of stage I ovarian mucinous tumors according to expansile or infiltrative type. *International Journal of Gynecologic Cancer*.

[28] Long J., Zhang L., Wan X. (2018). A four-gene-based prognostic model predicts overall survival in patients with hepatocellular carcinoma. *Journal of Cellular and Molecular Medicine*.

[29] Tian X., Zhu X., Yan T. (2017). Recurrence-associated gene signature optimizes recurrence-free survival prediction of colorectal cancer. *Molecular Oncology*.

[30] Huang T., Wang G., Yang L. (2017). MiR-186 inhibits proliferation, migration, and invasion of non-small cell lung cancer cells by downregulating Yin Yang 1. *Cancer Biomarkers*.

[31] Yao K., He L., Gan Y., Zeng Q., Dai Y., Tan J. (2015). MiR-186 suppresses the growth and metastasis of bladder cancer by targeting NSBP1. *Diagnostic Pathology*.

[32] Ries J., Baran C., Wehrhan F. (2017). Prognostic significance of altered miRNA expression in whole blood of OSCC patients. *Oncology Reports*.

[33] Zhu X., Shen H., Yin X. (2016). miR-186 regulation of Twist1 and ovarian cancer sensitivity to cisplatin. *Oncogene*.

[34] Hua X., Xiao Y., Pan W. (2016). miR-186 inhibits cell proliferation of prostate cancer by targeting GOLPH3. *American Journal of Cancer Research*.

[35] Li J., Xia L., Zhou Z. (2018). MiR-186-5p upregulation inhibits proliferation, metastasis and epithelial-to-mesenchymal transition of colorectal cancer cell by targeting ZEB1. *Archives of Biochemistry and Biophysics*.

[36] Porkka K. P., Helenius M. A., Visakorpi T. (2002). Cloning and characterization of a novel six-transmembrane protein STEAP2, expressed in normal and malignant prostate. *Laboratory Investigation*.

[37] Burnell S. E. A., Spencer-Harty S., Howarth S. (2019). Utilisation of the STEAP protein family in a diagnostic setting may provide a more comprehensive prognosis of prostate cancer. *PLoS One*.

[38] Whiteland H., Spencer-Harty S., Morgan C. (2014). A role for STEAP2 in prostate cancer progression. *Clinical & Experimental Metastasis*.

[39] Burnell S. E. A., Spencerharty S., Howarth S. (2018). STEAP2 knockdown reduces the invasive potential of prostate cancer cells. *Scientific Reports*.

[40] Hong L., Wang Y., Chen W., Yang S. (2018). MicroRNA-508 suppresses epithelial-mesenchymal transition, migration, and invasion of ovarian cancer cells through the MAPK1/ERK signaling pathway. *Journal of Cellular Biochemistry*.

[41] Wang W., Hu W., Wang Y., Yang J., Yue Z. (2019). MicroRNA-508 is downregulated in clear cell renal cell carcinoma and targets ZEB1 to suppress cell proliferation and invasion. *Experimental and Therapeutic Medicine*.

[42] Li R., Zhang L., Qin Z. (2019). High LINC00536 expression promotes tumor progression and poor prognosis in bladder cancer. *Experimental Cell Research*.

[43] Nam E. J., Kim S., Lee T. S. (2016). Primary and recurrent ovarian high-grade serous carcinomas display similar microRNA expression patterns relative to those of normal ovarian tissue. *Oncotarget*.

[44] Sun Y., Hu L., Zheng H. (2015). MiR-506 inhibits multiple targets in the epithelial-to-mesenchymal transition network and is associated with good prognosis in epithelial ovarian cancer. *The Journal of Pathology*.

[45] Liu G., Sun Y., Ji P. (2014). MiR-506 suppresses proliferation and induces senescence by directly targeting the CDK4/6-FOXM1 axis in ovarian cancer. *The Journal of Pathology*.

[46] Mo X., Wu Y., Chen L. (2019). Global expression profiling of metabolic pathway-related lncRNAs in human gastric cancer and the identification of RP11-555H23.1 as a new diagnostic biomarker. *Journal of Clinical Laboratory Analysis*.

[47] Hou W., Tang Q., Bi F. (2019). Comprehensive analysis of the aberrantly expressed profiles of lncRNAs, miRNAs and the regulation network of the associated ceRNAs in clear cell renal cell carcinoma. *Sheng Wu Yi Xue Gong Cheng Xue Za Zhi*.

[48] Chen H., Cai Y., Zheng Z., Chen Z., Xie W. (2018). Clinical significance of LINC0047 in renal cell carcinoma and its effect on 786-O cells. *Lingnan Modern Clinics in Surgery*.

[49] Zhang D. L., Zhou H. G., Liu J., Liu J., Mao J. (2019). Effect of LncRNA LINC00475 on biological behaviors of glioma cells. *Journal of Third Military Medical University*.

[50] van den Boomen D. J. H., Timms R. T., Grice G. L. (2014). TMEM129 is a Derlin-1 associated ERAD E3 ligase essential for virus-induced degradation of MHC-I. *Proceedings of the National Academy of Sciences*.

[51] Leffers N., Lambeck A. J. A., de Graeff P. (2008). Survival of ovarian cancer patients overexpressing the tumour antigen p53 is diminished in case of MHC class I down-regulation. *Gynecologic Oncology*.

[52] Mariani A., Wang C., Oberg A. L. (2019). Genes associated with bowel metastases in ovarian cancer. *Gynecologic Oncology*.

[53] Yoon G., Park J. Y., Kim H. J. (2019). ARID3A positivity correlated with favorable prognosis in patients with residual rectal cancer after Neoadjuvant chemoradiotherapy. *Anticancer Research*.

[54] Ma Q., Yang Y., Feng D. (2015). MAGI3 negatively regulates Wnt/β-catenin signaling and suppresses malignant phenotypes of glioma cells. *Oncotarget*.

[55] Fan Y., Shi Y., Lin Z. (2019). miR-9-5p suppresses malignant biological behaviors of human gastric cancer cells by negative regulation of TNFAIP8L3. *Digestive Diseases and Sciences*.

[56] Lian K., Ma C., Hao C. (2017). TIPE3 protein promotes breast cancer metastasis through activating AKT and NF-κB signaling pathways. *Oncotarget*.

[57] Zhang H., Wang Y., Chen T. (2019). Aberrant activation of Hedgehog signalling promotes cell migration and invasion via matrix metalloproteinase-7 in ovarian cancer cells. *Journal of Cancer*.

[58] Sradhanjali S., Reddy M. M. (2018). Inhibition of pyruvate dehydrogenase kinase as a therapeutic strategy against cancer. *Current Topics in Medicinal Chemistry*.

[59] Yang J. D., Seol S.-Y., Leem S.-H. (2011). Genes associated with recurrence of hepatocellular carcinoma: integrated analysis by gene expression and methylation profiling. *Journal of Korean Medical Science*.

[60] Kikuchi J., Hori M., Iha H. (2019). Soluble SLAMF7 promotes the growth of myeloma cells via homophilic interaction with surface SLAMF7. *Leukemia*.

[61] Tsujioka T., Yokoi A., Itano Y. (2015). Five-aza-2′-deoxycytidine-induced hypomethylation of cholesterol 25-hydroxylase gene is responsible for cell death of myelodysplasia/leukemia cells. *Scientific Reports*.

[62] Jeong O. S., Chae Y. C., Jung H. (2016). Long noncoding RNA linc00598 regulates CCND2 transcription and modulates the G1 checkpoint. *Scientific Reports*.

